# Detection of tumor‐derived cell‐free DNA from colorectal cancer peritoneal metastases in plasma and peritoneal fluid

**DOI:** 10.1002/cjp2.207

**Published:** 2021-02-26

**Authors:** Iris van't Erve, Koen P Rovers, Alexander Constantinides, Karen Bolhuis, Emma CE Wassenaar, Robin J Lurvink, Clément J Huysentruyt, Petur Snaebjornsson, Djamila Boerma, Daan van den Broek, Tineke E Buffart, Max J Lahaye, Arend GJ Aalbers, Niels FM Kok, Gerrit A Meijer, Cornelis JA Punt, Onno Kranenburg, Ignace HJT de Hingh, Remond JA Fijneman

**Affiliations:** ^1^ Department of Pathology The Netherlands Cancer Institute Amsterdam The Netherlands; ^2^ Department of Surgery Catharina Cancer Institute Eindhoven The Netherlands; ^3^ Department of Surgical Oncology, Division of Imaging and Cancer UMC Utrecht Utrecht The Netherlands; ^4^ Department of Medical Oncology Amsterdam University Medical Centers Amsterdam The Netherlands; ^5^ Department of Surgery St. Antonius Hospital Nieuwegein The Netherlands; ^6^ Department of Pathology Laboratory for Pathology and Medical Microbiology (PAMM) Eindhoven The Netherlands; ^7^ Department of Laboratory Medicine The Netherlands Cancer Institute Amsterdam The Netherlands; ^8^ Department of Gastrointestinal Oncology The Netherlands Cancer Institute Amsterdam The Netherlands; ^9^ Department of Radiology The Netherlands Cancer Institute Amsterdam The Netherlands; ^10^ Department of Surgery The Netherlands Cancer Institute Amsterdam The Netherlands; ^11^ Julius Center for Health Sciences and Primary Care University Medical Center Utrecht Utrecht The Netherlands; ^12^ Utrecht Platform for Organoid Technology Utrecht University Utrecht The Netherlands; ^13^ Department of Epidemiology, GROW‐School for Oncology and Developmental Biology Maastricht University Maastricht The Netherlands

**Keywords:** colorectal neoplasms, liquid biopsy, circulating tumor DNA, peritoneum, ascitic fluid, plasma, biomarkers

## Abstract

Tumor‐derived cell‐free DNA (cfDNA) is an emerging biomarker for guiding the personalized treatment of patients with metastatic colorectal cancer (CRC). While patients with CRC liver metastases (CRC‐LM) have relatively high levels of plasma cfDNA, little is known about patients with CRC peritoneal metastases (CRC‐PM). This study evaluated the presence of tumor‐derived cfDNA in plasma and peritoneal fluid (i.e. ascites or peritoneal washing) in 20 patients with isolated CRC‐PM and in the plasma of 100 patients with isolated CRC‐LM. Among tumor tissue *KRAS/BRAF* mutation carriers, tumor‐derived cfDNA was detected by droplet digital polymerase chain reaction (ddPCR) in plasma of 93% of CRC‐LM and 20% of CRC‐PM patients and in peritoneal fluid in all CRC‐PM patients. Mutant allele fraction (MAF) and mutant copies per ml (MTc/ml) were lower in CRC‐PM plasma than in CRC‐LM plasma (median MAF = 0.28 versus 18.9%, *p* < 0.0001; median MTc/ml = 21 versus 1,758, *p* < 0.0001). Within patients with CRC‐PM, higher cfDNA levels were observed in peritoneal fluid than in plasma (median MAF = 16.4 versus 0.28%, *p* = 0.0019; median MTc/ml = 305 versus 21, *p* = 0.0034). These data imply that tumor‐derived cfDNA in plasma is a poor biomarker to monitor CRC‐PM. Instead, cfDNA detection in peritoneal fluid may offer an alternative to guide CRC‐PM treatment decisions.

## Introduction

The peritoneum is a common and underdiagnosed metastatic site of colorectal cancer (CRC) [1, 2, 3]. Although patients with CRC peritoneal metastases (CRC‐PM) have a poor prognosis [1, 2], those with limited disease could achieve long‐term survival or cure after peritoneal cytoreductive surgery with or without hyperthermic intraperitoneal chemotherapy [4]. However, due to the extent of the disease, the majority of patients with peritoneal metastases receive systemic treatment instead [5, 6]. When systemic treatment is offered, treatment response evaluation may be impeded by poor visibility of peritoneal metastases on radiological imaging [7]. Therefore, there is a clinical need to detect peritoneal spread earlier and monitor treatment response better in patients with CRC‐PM.

In general, tumor‐derived cell‐free DNA (cfDNA) in plasma has great potential for tumor detection and monitoring of response to (targeted) therapies [8]. Yet, clinical implementation requires thorough validation for each specific clinical need. While patients with CRC liver metastases (CRC‐LM) are known to have relatively high levels of tumor‐derived cfDNA in plasma, little is known about cfDNA levels in patients with CRC‐PM [9, 10]. Therefore, the present study compared plasma cfDNA levels between patients with extensive isolated CRC‐PM and patients with extensive isolated CRC‐LM. Moreover, proximal fluids derived from the extracellular surroundings of tissue have been shown to be a useful liquid biopsy source of cfDNA, e.g. cerebrospinal fluid for brain cancer [11] and cerebrospinal fluid, pleural effusion, and ascites for non‐small‐cell lung cancer and melanoma patients [12]. More recently, peritoneal fluid was reported as a suitable liquid biopsy source for the detection of tumor‐derived cfDNA for peritoneal surface malignancies [13]. These studies illustrate the feasibility and indicate the putative clinical potential of using peritoneal fluid or ascites as a liquid biopsy source to detect tumor‐derived cfDNA. However, direct comparison of the detection of tumor‐derived cfDNA in peritoneal fluid or ascites to plasma in patients with isolated CRC‐PM has not been reported. In the present study, tumor‐derived cfDNA levels were also compared between peritoneal fluid and plasma in patients with CRC‐PM to explore peritoneal fluid as a potential source of cfDNA in this patient group.

## Materials and methods

Blood from patients with histologically proven CRC with isolated, initially unresectable liver metastases was collected in the multicenter CAIRO5 trial (NCT02162563) [14]. Blood and peritoneal fluid were obtained from patients with histologically proven isolated and unresectable CRC‐PM, participating in the CRC‐PIPAC trial (NCT03246321) [15]. Both trials were approved by a medical ethical committee, and all patients signed written informed consent for study participation, as well as liquid biopsy and tumor tissue collection for translational research. The liquid biopsies (i.e. blood and peritoneal fluid) were collected prior to study treatment, processed, and stored centrally (see supplementary material, Supplementary materials and methods). Peritoneal fluid (i.e. ascites or a peritoneal washing with saline if ascites was not present) in the CRC‐PIPAC trial was obtained during the initial laparoscopy. Mutation analysis of tumor tissue was performed in all enrolled patients (see supplementary material, Supplementary materials and methods). Patients were selected for cfDNA analysis when *KRAS* or *BRAF* mutations were found in their tumor tissue. In brief, cfDNA from plasma and peritoneal fluid was isolated using the QIAsymphony (Qiagen, Düsseldorf, Germany) and analyzed by droplet digital polymerase chain reaction (ddPCR; Bio‐Rad, Hercules, CA, USA). Levels of cfDNA were measured and presented as mutant allele fraction (MAF) and mutant copies per ml input (MTc/ml) and compared between groups using a Mann–Whitney *U*‐test with a two‐sided *P*‐value of 0.05 as a cut‐off for significance (see supplementary material, Supplementary materials and methods).

## Results

To investigate tumor‐derived cfDNA using sensitive and validated PCR‐based assays, we focused on the detection of *KRAS* and *BRAF* mutations. Of 100 patients with isolated CRC‐LM (Table 1), 57 (57%) had a *KRAS* or *BRAF* tumor tissue mutation (see supplementary material, Table S1). Of these, 46 (81%) had synchronous metastases and 32 (56%) had an unresected primary tumor at the time of liquid biopsy collection. Of 20 patients with isolated CRC‐PM (Table 1), 11 (55%) had a *KRAS* or *BRAF* tumor tissue mutation and were selected for cfDNA analysis (see supplementary material, Table S2). Of these, seven (64%) had synchronous metastases with an unresected primary tumor at the time of liquid biopsy collection.

**Table 1:** Summary of baseline characteristics of patients with isolated CRC‐LM enrolled in the CAIRO5 trial and patients with isolated CRC‐PM enrolled in the CRC‐PIPAC trial.

Characteristic	CRC‐LM cohort (*N =* 100)	CRC‐PM cohort (*N =* 20)
Age at inclusion (years, mean ± SD)	60 ± 10	63 ± 9.8
Sex (*N* [%])		
Male	64 (64)	12 (60)
Female	36 (36)	8 (40)
Primary tumor (*N* [%])		
Resected	45 (45)	6 (30)
Unresected	55 (55)	14 (70)
Metastases (*N* [%])		
Synchronous	82 (82)	15 (75)
Metachronous	18 (18)	5 (25)
Source of tumor tissue mutation analysis (*N* [%])		
Primary tumor	91 (91)	5 (25)
Metastases	9 (9)	2 (10)
Both	0 (0)	13 (65)
Tumor tissue mutation (*N* [%])		
*KRAS*	54 (54)	9 (45)
*BRAF*	3 (3)	2 (10)
No mutation detected	43 (43)	9 (45)
Systemic therapy <6 months before study registration (*N* [%])		
Yes	0 (0)	11 (55)
No	100 (100)	9 (45)

**Figure 1:**
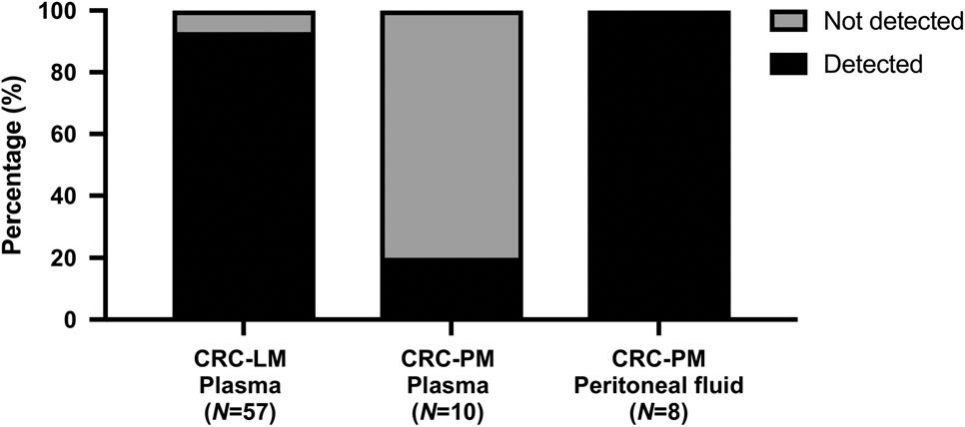
Among patients with a tumor tissue *KRAS/BRAF* mutation, the percentage of patients in whom a *KRAS/BRAF* mutation was also detected: (left) in plasma of patients with isolated CRC‐LM; (middle) in plasma of patients with isolated CRC‐PM; and (right) in peritoneal fluid of patients with isolated CRC‐PM.

**Figure 2:**
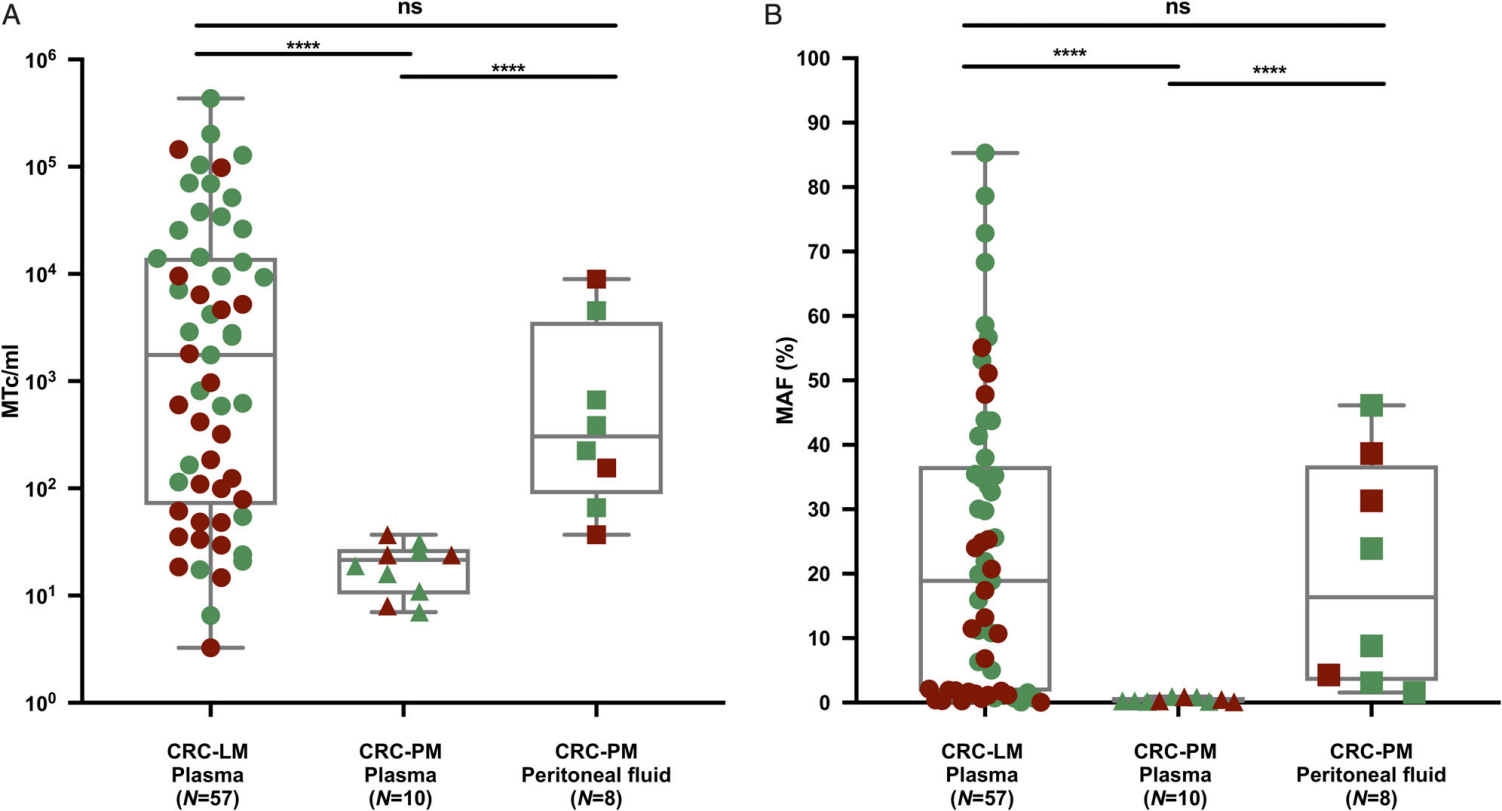
(A) MTc/ml plasma or peritoneal fluid and (B) MAF measured in plasma of patients with isolated CRC‐LM (*N =* 57), in plasma of patients with isolated CRC‐PM (*N =* 10), and in peritoneal fluid of patients with isolated CRC‐PM (*N =* 8). Red symbols: patients with a resected primary tumor at the time of blood and peritoneal fluid collection. Green symbols: patients with an unresected primary tumor at the time of blood and peritoneal fluid collection. ns, Not significant; ****p < 0.0001.

Among the patients with a *KRAS/BRAF* tumor tissue mutation, *KRAS/BRAF* mutations were detected in plasma cfDNA in 93% of patients with CRC‐LM compared to 20% of patients with CRC‐PM (*p* < 0.0001; Figure 1). In contrast, these mutations could be detected in all available peritoneal fluid samples from the same CRC‐PM patients (Figure 1). Both the MAF and MTc/ml plasma cfDNA values were lower in patients with CRC‐PM than in patients with CRC‐LM (MAF: 0.28% [0.12–0.92%] versus 18.9% [0.06–85.3%], *p* < 0.0001; MTC/ml: 21 [7–37] versus 1,758 [3–432.563], *p* < 0.0001; Figure 2). In patients with CRC‐PM, both the MAF and MTc/ml cfDNA values were higher in peritoneal fluid than in plasma (MAF: 16.4% [1.56–46.1%] versus 0.28 [0.12–0.92%], *p* < 0.0001; MTc/ml: 305 [36–8,947] versus 21 [7–37], *p* < 0.0001; Figure 2).

The presence of the primary tumor at the time of liquid biopsy collection likely affects cfDNA levels in plasma and peritoneal fluid. Indeed, compared to CRC‐LM patients with a resected primary tumor, CRC‐LM patients with an unresected primary tumor had higher MAF (*p* = 0.0019) and MTc/ml (*p* = 0.0034) plasma cfDNA levels. Interestingly, no such difference in the MAF or MTc/ml was found in patients with CRC‐PM between resected or unresected primary tumors, neither for plasma nor for peritoneal fluid (Figure 2). These data suggest that, in addition to the CRC‐PM, the primary tumors of patients with isolated CRC‐PM also have a low propensity to shed cfDNA into the circulation.

## Discussion

The propensity of a tumor to shed cfDNA into the circulation varies among cancer types and is known to be relatively high for patients with metastatic CRC [16]. However, differences between CRC metastatic sites in general, and analyses of CRC‐PM in particular, have not been investigated extensively and require studies of carefully selected patient populations with isolated metastases. The present study demonstrates that plasma cfDNA is not a sensitive biomarker to detect isolated CRC‐PM, in sharp contrast to patients with isolated CRC‐LM.

The low levels of plasma cfDNA in CRC‐PM relative to CRC‐LM could be explained by several mechanisms. Peritoneal metastases and liver metastases originate from different dissemination patterns of the primary tumor. While dissemination to the liver mainly occurs hematogenously via the portal vein, peritoneal dissemination is characterized either by peritoneal penetration from the primary tumor or iatrogenic spread as a consequence of incomplete resection [17]. These intrinsic differences in dissemination pathways of the primary tumor may be associated with differences in the propensity to shed cfDNA into the circulation, which might explain why a confounding effect of the presence of the primary tumor on cfDNA plasma levels was not observed for patients with CRC‐PM but was present in patients with CRC‐LM (Figure 2). Moreover, the peritoneum–plasma barrier may restrict cfDNA release from peritoneal metastases into the systemic circulation [18]. The low levels of plasma cfDNA in patients with isolated CRC‐PM may indicate that peritoneal metastases are a locoregional rather than a systemic disease. This supports the general assumption that palliative systemic therapy is relatively less effective for isolated CRC‐PM than for isolated nonperitoneal colorectal metastases [19]. Therefore, plasma cfDNA does not appear to be a useful biomarker to detect CRC‐PM in an early operable stage or to monitor treatment response in this particular patient group. Theoretically, when tumor‐derived cfDNA is detected in the plasma of patients with presumably isolated CRC‐PM, this may be indicative of occult hematogenous metastases and future systemic disease progression. Consequently, patients who qualify for peritoneal cytoreductive surgery and who test positive for plasma cfDNA might benefit from perioperative systemic therapy, a hypothesis that is currently being tested in the multicenter CAIRO6 clinical trial [20].

The high detectability of cfDNA in peritoneal fluid of all patients with isolated CRC‐PM suggests that peritoneal fluid may serve as a more useful source of cfDNA than plasma for this patient group. As ascites is not (abundantly) present in all patients with peritoneal metastases and mostly manifests itself in a late stage of the disease, cfDNA analysis of peritoneal washes may offer an alternative to detect and monitor peritoneal spread in patients with CRC‐PM. Peritoneal washing could be a low‐risk minimally invasive method in patients with isolated CRC‐PM when laparoscopy is already part of clinical care, i.e. laparoscopy as part of follow‐up in patients with high‐risk (i.e. pT4a‐bN0‐2M0) CRC after resection [21]. Aspiration of ascites or peritoneal washing could be a method to obtain cfDNA for molecular profiling, response monitoring, and detection of chemotherapy resistance in patients with isolated CRC‐PM. This hypothesis is currently being tested in the multicenter INTERACT trial [22] and may also apply to other malignancies that frequently metastasize to the peritoneum, especially those that are difficult to access for histological biopsy, such as ovarian, pancreatic, appendiceal, and small bowel cancers.

This study has several limitations. First, the number of patients within the CRC‐PM cohort was relatively small. Second, cfDNA analyses were restricted to a subset of patients with *KRAS/BRAF* hot‐spot mutations. In addition, while the aim was to measure cfDNA derived from metastases, a subgroup of the patients still had an unresected primary tumor that may have contributed to the cfDNA signals measured. Finally, in the present study, some isolated CRC‐PM were previously treated with systemic therapy, whereas all isolated CRC‐LM were previously untreated at least 6 months before study registration. Despite these limitations, clear differences were observed between the CRC‐PM and CRC‐LM cohorts.

In conclusion, cfDNA in plasma is becoming a realistic approach to guide personalized treatment of patients with CRC‐LM, while this approach appears less suitable for patients with CRC‐PM, who instead may benefit from the detection of tumor‐derived cfDNA in peritoneal fluid. The observations in this study underscore the biological relevance of the differences in growth patterns between systemic and peritoneal metastases, which should be taken into account when considering the clinical utility of cfDNA.

## Author contributions statement

IvE, OK and RJAF are responsible for the concept and design of the study. Funding was obtained by CJAP, IHJTdH and RJAF. Patient acquisition and material collection were performed by KPR, AC, KB, ECEW and RJL. IvE performed the analyses and drafted the manuscript under the supervision of GAM, CJAP, IHJTdH and RJAF. All authors were involved in reviewing the manuscript for important intellectual content.

## Supporting information


**Supplementary materials and methods**
**Table S1.** Overview of individual patients with isolated CRC‐LM with a *KRAS* or *BRAF* mutation in tumor tissue
**Table S2.** Overview of individual patients with isolated CRC‐PM with a *KRAS* or *BRAF* mutation in tumor tissue
