# Supplementary material for: Detection of tumor‐derived cell‐free DNA from colorectal cancer peritoneal metastases in plasma and peritoneal fluid
Source: J Pathol Clin Res. 2021 Feb 26;7(3):203–8. doi: 10.1002/cjp2.207 (PMC8073000; doi:10.1002/cjp2.207)
Supplement: Supplementary file 1 — Supplementary materials and methods Table S1. Overview of individual patients with isolated CRC‐LM with a KRAS or BRAF mutation in tumor tissue Table S2. Overview of individual patients with isolated CRC‐PM with a KRAS or BRAF mutation in tumor tissue [file CJP2-7-203-s001.docx]

**Detection of tumor-derived cell-free DNA from colorectal cancer peritoneal metastases in plasma and peritoneal fluid**

I van ‘t Erve *et al*. *J Pathol Clin Res* DOI: 10.1002/cjp2.207

**Supplementary Material**

**Supplementary Materials and Methods**

**Table S1.** Overview of individual patients with isolated CRC-LM with a *KRAS* or *BRAF* mutations in tumor tissue

**Table S2**. Overview of individual patients with isolated CRC-PM with a *KRAS* or *BRAF* mutation in tumor tissue

**Supplementary Materials and Methods**

Subjects

Blood was collected between February 2015 and September 2018 from 100 patients with extensive CRC-LM participating in the CAIRO5 multi-center clinical trial. Patients with a histologically and radiologically proven colorectal cancer with initially unresectable isolated liver metastases that were previously untreated were included in this study. Blood and peritoneal fluid were obtained between October 2017 and December 2018 from 20 patients with extensive CRC-PM (Peritoneal Cancer Index >20) participating in the CRC-PIPAC trial in two Dutch tertiary referral hospitals. Patients with a histologically and radiologically proven colorectal cancer and initially unresectable isolated peritoneal metastases, not previously treated with pressurized intraperitoneal aerosol chemotherapy (PIPAC) were included in this study. Patients in both studies had a WHO performance status of 0 or 1 and no contraindication for the planned intervention.

Tumor tissue mutation analyses

In the CAIRO5 trial, the *KRAS*/*BRAF* tumor tissue mutation status of the CRC-LM was assessed on formalin-fixed paraffin embedded material of the primary tumor or a liver metastasis as part of the trial protocol. In the CRC-PIPAC trial, the *KRAS*/*BRAF* tumor tissue mutation analysis of the primary tumor was extracted from pathology reports. Additionally, CRC-PM were biopsied and formalin-fixed paraffin-embedded prior to study treatment as part of the CRC-PIPAC trial protocol. DNA of these samples was isolated from five to ten 10 μm slides, using the Qiagen AllPrep DNA/RNA/miRNA Universal kit (Qiagen, Düsseldorf, Germany). Subsequently, the *KRAS* and *BRAF* mutation status was assessed by Sequenom MassARRAY (Agena Bioscience, Hamburg, Germany) using a small multigene panel following the standard diagnostics workflow. The panel consisted of 67 hotspots in 8 genes (*KRAS, NRAS, BRAF, PIK3CA, MAP2K1, AKT, DDR2, EGFR*).

Collection, processing, and storage of liquid biopsies

In both trials, blood was collected after trial enrolment and prior to study treatment in the hospital of enrolment using 10 ml cell-free DNA BCT^®^ tubes (Streck, La Vista, USA). Collected blood samples were shipped to the Netherlands Cancer Institute, Amsterdam, the Netherlands. Here, plasma was obtained after a two-step centrifugation process (10 minutes at 1700g followed by 10 minutes at 20 000g) and stored at -80°C until further processing. In the CRC-PIPAC trial, the peritoneal fluid was obtained during the initial laparoscopy, before administration of the intraperitoneal chemotherapy. When ascites was present, it was completely evacuated and 5 ml of ascites was collected in Nunc^®^ CryoTubes^®^ (Merck, Darmstadt, Germany). Approximately 20 minutes after collection, the ascites was centrifugated two times 5 minutes at 420g before the supernatant was snap frozen and stored at -80°C until further processing. When ascites was absent, a peritoneal washing was performed by rinsing the abdominal cavity with 50 ml 0.9% NaCl, after which 5 ml of the collected peritoneal washing was processed identically to the ascites.

Isolation of cfDNA

A total of 60 μl of cell-free DNA (cfDNA) of every sample of plasma and peritoneal fluid was isolated using the QIAsymphony (Qiagen, Düsseldorf, Germany) and stored at 4°C until analysis. Concentration of the cfDNA was measured using the Qubit™ dsDNA High-Sensitivity Fluorometer Kit (TFS, Waltham, USA), and ranged from 0.12 to 36.6 ng/μl.

Mutation analysis of cfDNA

Mutation analysis of cfDNA was performed by droplet digital PCR (Bio-Rad, Hercules, USA) using the ddPCR™ KRAS G12/13, KRAS Q61 or BRAF V600 Screening Kits according to the manufacturer’s instructions. All measurements were performed in duplicate and included a blank (nuclease free water) and an in-house positive control. Data were analyzed using the QuantaSoft^TM^ software version 1.6.6 (Bio-Rad, Hercules, USA). Individual wells with less than 10 000 total events (droplets) were excluded from the analysis. The limit of detection was determined based on the limit of blank, adjusting the outcome according to a predefined ratio of false positive mutants found in wildtype samples. For the mutant cases, mutant allele fraction (MAF) and mutant copies per ml input (MTc/ml) were assessed.

Statistical analyses

Based on normality testing, data were presented as mean and standard deviation or as median with the corresponding minimum to maximum range. The number of patients with detectable plasma cfDNA mutations was compared between groups using a chi-square test. The MAF and MTc/ml plasma cfDNA levels were compared across groups using a Mann-Whitney-U test. Statistical analyses were performed with Prism version 8 (GraphPad Software, Inc., USA), with a two-sided P-value of 0.05 as a cut-off value for significance.

**Table S1.** Overview of individual patients with isolated CRC-LM with a *KRAS* or *BRAF* mutations in tumor tissue

| **ID** | **CRC metastases** | **Primary tumor resected** | **Source tumor tissue mutation analysis** | ***KRAS/BRAF* mutation tumor tissue** | **Bio-Rad screening kit** | **Mutation detected in plasma cfDNA** | **Plasma MAF (%)** | **Plasma MTc/ml** |
| --- | --- | --- | --- | --- | --- | --- | --- | --- |
| 1 | Synchronous | Yes | Primary tumor | KRAS A146T | #10049550 | Yes | 51.12% | 144683 |
| 2 | Synchronous | Yes | Primary tumor | KRAS G12A | #1863506 | Yes | 11.48% | 418 |
| 3 | Synchronous | Yes | Primary tumor | KRAS Q61H | #12001626 | Yes | 1.65% | 79 |
| 4 | Synchronous | Yes | Primary tumor | BRAF V600E | #12001037 | Yes | 24.00% | 4644 |
| 5 | Synchronous | No | Primary tumor | KRAS G12V | #1863506 | Yes | 37.98% | 34203 |
| 6 | Synchronous | Yes | Primary tumor | BRAF V600E | #12001037 | Yes | 0.31% | 15 |
| 7 | Synchronous | No | Primary tumor | KRAS G12D | #1863506 | Yes | 18.88% | 26313 |
| 8 | Synchronous | No | Primary tumor | BRAF V600E | #12001037 | Yes | 10.83% | 586 |
| 9 | Synchronous | Yes | Primary tumor | KRAS G12D | #1863506 | No |  |  |
| 10 | Synchronous | No | Primary tumor | KRAS G12V | #1863506 | Yes | 33.59% | 4199 |
| 11 | Synchronous | No | Primary tumor | KRAS G12D | #1863506 | Yes | 35.21% | 12905 |
| 12 | Synchronous | No | Primary tumor | KRAS A146T | #10049550 | Yes | 43.75% | 70483 |
| 13 | Synchronous | No | Liver metastasis | KRAS G12C | #1863506 | Yes | 53.12% | 103847 |
| 14 | Metachronous | Yes | Primary tumor | KRAS G12C | #1863506 | Yes | 1.15% | 48 |
| 15 | Synchronous | Yes | Primary tumor | KRAS G12V | #1863506 | Yes | 55.10% | 9569 |
| 16 | Synchronous | No | Primary tumor | KRAS G12C | #1863506 | Yes | 30.04% | 69456 |
| 17 | Synchronous | No | Primary tumor | KRAS G12C | #1863506 | Yes | 56.69% | 2894 |
| 18 | Metachronous | Yes | Primary tumor | KRAS G12V | #1863506 | Yes | 1.78% | 185 |
| 19 | Metachronous | Yes | Primary tumor | KRAS G13D | #1863506 | Yes | 0.41% | 18 |
| 20 | Synchronous | No | Primary tumor | KRAS G12A | #1863506 | Yes | 41.39% | 51438 |
| 21 | Synchronous | No | Primary tumor | KRAS G12C | #1863506 | No |  |  |
| 22 | Metachronous | Yes | Primary tumor | KRAS Q61L | #12001626 | Yes | 1.18% | 30 |
| 23 | Synchronous | Yes | Primary tumor | KRAS Q61R | #12001626 | Yes | 20.73% | 49 |
| 24 | Metachronous | Yes | Primary tumor | KRAS G13A | #1863506 | Yes | 1.84% | 124 |
| 25 | Synchronous | No | Liver metastasis | KRAS A146T | #10049550 | Yes | 78.62% | 127859 |
| 26 | Synchronous | No | Primary tumor | KRAS A146T | #10049550 | Yes | 72.85% | 201346 |
| 27 | Synchronous | No | Primary tumor | KRAS G12V | #1863506 | Yes | 6.35% | 165 |
| 28 | Synchronous | No | Liver metastasis | KRAS A146T | #10049550 | Yes | 85.29% | 432563 |
| 29 | Synchronous | No | Primary tumor | KRAS G12D | #1863506 | Yes | 1.53% | 55 |
| 30 | Synchronous | Yes | Primary tumor | KRAS G12D | #1863506 | Yes | 47.83% | 98046 |
| 31 | Synchronous | No | Primary tumor | KRAS G12D | #1863506 | No |  |  |
| 32 | Synchronous | No | Primary tumor | KRAS G12S | #1863506 | Yes | 19.93% | 622 |
| 33 | Synchronous | No | Primary tumor | KRAS G12D | #1863506 | Yes | 32.69% | 37832 |
| 34 | Synchronous | No | Primary tumor | KRAS G12A | #1863506 | Yes | 34.80% | 9321 |
| 35 | Metachronous | Yes | Primary tumor | KRAS G12V | #1863506 | Yes | 25.30% | 5218 |
| 36 | Synchronous | No | Liver metastasis | KRAS G12V | #1863506 | Yes | 35.46% | 13909 |
| 37 | Synchronous | No | Primary tumor | KRAS G12V | #1863506 | Yes | 0.73% | 17 |
| 38 | Synchronous | No | Primary tumor | KRAS G12D | #1863506 | Yes | 5.02% | 115 |
| 39 | Synchronous | No | Primary tumor | KRAS G13D | #1863506 | Yes | 43.84% | 25553 |
| 40 | Synchronous | Yes | Primary tumor | KRAS G12D | #1863506 | No |  |  |
| 41 | Synchronous | Yes | Primary tumor | KRAS G12V | #1863506 | Yes | 13.18% | 969 |
| 42 | Synchronous | No | Primary tumor | KRAS EXON2 | #1863506 | Yes | 29.77% | 2624 |
| 43 | Metachronous | Yes | Liver metastasis | KRAS G12V | #1863506 | Yes | 2.10% | 110 |
| 44 | Synchronous | No | Liver metastasis | KRAS G12V | #1863506 | Yes | 25.61% | 7073 |
| 45 | Synchronous | No | Primary tumor | KRAS A146T | #10049550 | Yes | 11.20% | 1758 |
| 46 | Synchronous | No | Primary tumor | KRAS G12D | #1863506 | Yes | 21.94% | 2794 |
| 47 | Synchronous | No | Primary tumor | KRAS A146T | #10049550 | Yes | 0.09% | 7 |
| 48 | Synchronous | No | Primary tumor | KRAS G12A | #1863506 | Yes | 58.58% | 14363 |
| 49 | Synchronous | Yes | Primary tumor | KRAS G12S | #1863506 | Yes | 24.86% | 6402 |
| 50 | Synchronous | Yes | Primary tumor | KRAS G12V | #1863506 | Yes | 6.83% | 320 |
| 51 | Metachronous | Yes | Primary tumor | KRAS G12D | #1863506 | Yes | 10.72% | 600 |
| 52 | Metachronous | Yes | Primary tumor | KRAS G12D | #1863506 | Yes | 1.35% | 61 |
| 53 | Synchronous | No | Primary tumor | KRAS G12A | #1863506 | Yes | 15.92% | 812 |
| 54 | Metachronous | Yes | Primary tumor | KRAS G12R | #1863506 | Yes | 17.42% | 1798 |
| 55 | Metachronous | Yes | Primary tumor | KRAS EXON4 | #10049550 | Yes | 0.06% | 3 |
| 56 | Synchronous | Yes | Liver metastasis | KRAS G12V | #1863506 | Yes | 1.94% | 100 |
| 57 | Synchronous | No | Primary tumor | KRAS A146T | #10049550 | Yes | 68.33% | 9510 |

*Abbreviations: MAF = mutant allele frequency, MTc/ml = mutant copies per ml input, CRC-LM = colorectal cancer liver metastases*

**Table S2**. Overview of individual patients with isolated CRC-PM with a *KRAS* or *BRAF* mutation in tumor tissue

| **ID** | **CRC metastases** | **Primary tumor resected** | **Systemic therapy^1^** | **Source tumor tissue mutation analysis** | ***KRAS/BRAF* mutation tumor tissue** | **Bio-Rad screening kit** | **Mutation detected in plasma cfDNA** | **Plasma MAF (%)** | **Plasma MTc/ml** | **Mutation detected in peritoneal fluid cfDNA** | **Peritoneal fluid MAF (%)** | **Peritoneal fluid MTc/ml** | **Type of peritoneal fluid** |
| --- | --- | --- | --- | --- | --- | --- | --- | --- | --- | --- | --- | --- | --- |
| 1 | Synchronous | No | Yes | PT and PM | KRAS Q61H | #12001626 | No |  |  | NA | NA | NA | NA |
| 2 | Metachronous | Yes | Yes | PT and PM | BRAF V600E | #12001037 | Yes | 0.12% | 8 | Yes | 4.27% | 37 | Washing |
| 3 | Metachronous | Yes | No | PT and PM | KRAS G13D | #1863506 | No |  |  | NA | NA | NA | NA |
| 4 | Synchronous | No | No | PT | KRAS G12V | #1863506 | No |  |  | Yes | 1.56% | 224 | Ascites |
| 5 | Metachronous | Yes | Yes | PT and PM | KRAS G13D | #1863506 | No |  |  | Yes | 31.33% | 155 | Ascites |
| 6 | Synchronous | No | No | PT and PM | KRAS 12/13 | #1863506 | No |  |  | Yes | 3.09% | 66 | Ascites |
| 7 | Synchronous | No | Yes | PT and PM | KRAS G12V | #1863506 | No |  |  | Yes | 8.81% | 668 | Ascites |
| 8 | Synchronous | No | No | PM | KRAS G12D | #1863506 | Yes | 0.27% | 11 | Yes | 46.14% | 4545 | Ascites |
| 9 | Synchronous | No | Yes | PM | KRAS G12S | #1863506 | NA | NA | NA | Yes | 23.92% | 386 | Washing |
| 10 | Synchronous | No | Yes | PT and PM | KRAS G12S | #1863506 | No |  |  | NA | NA | NA | NA |
| 11 | Metachronous | Yes | No | PT and PM | BRAF V600E | #12001037 | No |  |  | Yes | 38.65% | 8947 | Ascites |

*Abbreviations: NA = Not Available, PT = primary tumor, PM = peritoneal metastasis, MAF = mutant allele frequency, MTc/ml = mutant copies per ml input, CRC-PM = colorectal cancer peritoneal metastases*

*^1^ Systemic therapy within 6 months before study registration*
